# Practical Observations on Mineral Waters and Baths, &c.

**Published:** 1847-04-01

**Authors:** 


### Practical Observations on Mineral Waters and Baths, with Notes of some Continental Climates, and a Re-print of the Cold Water Cure. By *Edwin Lee*, Esq. 12mo. pp. 134. Churchill, 1846.

We suppose no member of the profession in this country is so well acquainted with its actual state upon the Continent as the author of the little work before us. We have frequently derived advantage from the perusal of the accounts his somewhat prolific pen has furnished, and we can especially recommend the present production to the notice of our readers.

The practice of having recourse to the foreign mineral waters is, and is likely, in this age of railroads and augmented continental intercommunication, to continue to be an increasing one; and those who are consulted or advised by their patients previous to quitting home, should be made well aware that the mere recommendation to resort to the spas is too indefinite, the composition of their waters being so exceedingly various in its character as to render what might act beneficially in one case, a very dangerous remedy or an actual poison to the system in another. Few medical men have the opportunity of repairing to the Continent to examine into these matters for themselves, and therefore works like those of our respected friend the late editor of this Journal, and the present one by Mr. Lee, become of great utility in assisting us in giving useful advice. Mr. Lee moreover, long as he has been accustomed to a continental residence, has not returned with that enthusiastic and exclusive admiration for everything not to be found in this island, which is so ridiculous in some who have written, and in more who speak upon the subject. On the contrary, his remarks upon the limitations to the utility of the mineral waters are very discriminate and just, and although we think he has needlessly multiplied the extracts in their favour from the works of German writers, he seems fully aware that the native historians of particular spas are not credible witnesses, and that many cases treated by their agency are so inappropriately, and would have done as well or better without their assistance. We quite agree with him in his general observation, that the English, from their impatient habit of expecting to see marked results quickly follow the use of medicines, have never done justice to these medicinal agents; and that in a numerous class of chronic diseases they may prove, by their almost imperceptible modification of the various secretions of the economy, of immense benefit.

After several introductory observations, Mr. Lee arranges the various mineral waters into certain classes, the characteristics of which are derived from their chemical composition; and indicates the names of the chief spas whose waters can thus be brought under these respective heads. He divides them into 1, *Sulphureous.* 2. *Chalybeate.* 3. *Saline Thermal,* consisting chiefly of "muriate of soda in combination with earthy or alkaline sulphates, carbonates and muriates, small quantities of metal, and the animal substance termed glairine, and a a certain proportion of carbonic acid gas." 4. *Saline Aperient,* in which sulphate of soda or of magnesia is the predominating ingredient. 5. *Cold Salt, or Brine Springs.* 6. *Alkaline.* 7. *Acidulous Springs,* containing a large proportion of free carbonic acid. 8. *Slightly Mineralised Springs,* or, as they are called by some of the German writers, "chemically-indifferent springs."

"It will be seen, that although I have often mentioned the same diseases as likely to be benefited by different springs of the same class, and even by springs belonging to different classes, yet that some springs are more adapted to particular cases than others, even though they may appear to be very analogous. It is likewise true, that it may be said of several mineral waters, differing in their nature, (as of other therapeutic agents,) that they may be advantageously employed in similar diseases, abstractedly considered; and thus a sulphurous, a saline, or a chalybeate spring may be said, with truth, to be efficacious in scrofulous, bronchial, or rheumatic disease; but each of these and other classes of diseases varies so much in its nature in different individuals and under different circumstances, that it is only by studying the peculiarities in individual cases that the practitioner can best determine from which kind of spring most benefit is likely to be derived in any given case. Several mineral springs have for a long period enjoyed a special reputation, founded upon experience of their greater efficacy in certain classes of diseases. Thus, Ems and Caûterets have acquired a name for their powers in pulmonary affections; Carlsbad, Vichy, &c. for abdominal and liver derangement; Wiesbaden and Bourbonne les Bains, in rheumatic and paralytic complaints, and so forth; but it must not be inferred from this, that all pulmonary affections to which mineral waters are applicable, are necessarily to be sent to a spring resembling either Ems or Caûterets, nor that Weisbaden, Bourbonne, and similar waters, are to be preferred in all cases of rheumatism or paralysis; as experience demonstrates that, in some affections of the lungs and air-passages, the above-mentioned waters would be prejudicial, and in which a water of a very different kind would be likely to be extremely beneficial; and also, that many cases of rheumatism and paralysis would be much more benefited by other waters, differing very greatly in their composition from those of Bourbonne or Wiesbaden. In this manner, (viz. the stating that such or such a spring is good for any particular disease, without special enquiry into the pathological and constitutional peculiarities), much harm has been done, and many invalids have consequently returned home disappointed, or worse than they came; but even when a spring appears to the practitioner to be more particularly indicated, it would be unreasonable to expect that its administration is always to be attended with benefit, as various circumstances, such as the idiosyncracy of the patient, the state of the disease, error on the part of the practitioner in the recommendation and management of the course, or on the part of the patient as regards mode of living, &c., may occur to prevent a beneficial action." P. 63.

Dr. Lee gives a general view of the different diseased conditions of the economy which these various waters and baths are usually employed for the correction of; and to which we can refer practitioners who are about to send patients abroad for much useful information, especially as the author's practical acquaintance with the true characters of the various climates, enables him to add much incidental information upon that head. The diseases of the economy specified as those in which an intelligent recourse to mineral waters may prove useful are—1. Various chronic disordered states of the Digestive Organs, (the repleted condition of the abdominal venous system being, according to the German pathologists, a chief cause of many of the chronic diseases for which mineral waters prove the best remedies). 2. Gout and Rheumatism. 3. The Waters of Vichy in Calculous Disorders. 4. Scrofula. 5. Pulmonary Disease. 6. Chlorosis. 7. Various disordered states of the Nervous System. 8. Chronic Cutaneous Diseases. 9. Chronic Discharges, as Leucorrhœa, when not dependent on organic mischief. For these various conditions the different waters are pointed out with much discrimination.

Mr. Lee speaks of the utility of *artificial mineral waters* more favourably than many do, which is gratifying from so competent an observer, as these are so accessible at Brighton, within the compass of even a Cockney-trip. Their great expense, however, quite precludes their employment as baths, while in many affections the bathing is a far more essential means of cure than the mere ingurgitation of the water.

"When drinking is the more essential part of the treatment, artificial waters have, in some respects, the advantage over natural ones, such as being available during the greater part of the year, instead of their employment being restricted to a few months in the summer, as is the case at the various baths: they may also in some cases be used as a preparatory measure, or subsequent to the use of the natural springs. Several of the most powerful waters being collected together in one establishment, if one which appeared to be indicated did not suit, recourse might be had to another; at all events the disappointment would not be so great as where a person had been induced to make a journey of several hundred miles to a spring, and found it unsuited to his case; a circumstance of not unfrequent occurrence, and often depending upon the adoption of the advice of those who are but little acquainted with the properties and effects of the different springs, or who are prejudiced in favour of particular ones; though it must be admitted that patients occasionally suffer disappointment from the difficulty which even experienced practitioners have in forming an opinion, in obscure chronic cases, as the means most likely to be of service; from the intractableness or incurability of the complaints; from the idiosyncrasy of individuals, &c., in consequence of which the effects of a mineral spring cannot always be estimated before trial has been made; as is likewise seen to be the case with many remedies in the ordinary practice of medicine, when medicines apparently indicated disagree, or do not produce the effects anticipated." P. 121.

Mr. Lee completes the volume by stitching into it a re-print of his little *brochure* upon the Cold Water Cure. He is no enthusiastic admirer of its virtues, but freely admits the errors and mischief which have resulted from its so general adoption in Germany. With ourselves it is evidently on the wane. What is to be the next fashionable medical delusion?

